# FEASIBILITY OF USING AN E-INTERVENTION PLATFORM FOR LONGITUDINAL RESEARCH ON DEMENTIA CARE DYADS

**DOI:** 10.1093/geroni/igae098.3981

**Published:** 2024-12-31

**Authors:** Constanca Paul, Soraia Teles

**Affiliations:** University of Porto, Porto, Portugal; University of Porto, Porto, Portugal

## Abstract

Remote Measurement Technologies (RMT) are increasingly used in research, offering cost-effective and unobtrusive data collection. This study examines the feasibility of using the e-intervention “iSupport-Portugal,” adapted from WHO’s “iSupport” for longitudinal research on dementia care dyads. A prospective cohort study was designed to predict the institutionalization of persons with dementia (PwD) at 9 months using baseline data from informal caregivers (IC) registering on isupport-portugal.pt. Participation rates, loss to follow-up, and event rates were analyzed. Completers and non-completers of baseline and follow-up assessments were compared on sociodemographic and psychosocial variables. In 16 months, 253 IC registered on isupport-portugal.pt, with 82% agreeing to research. Most are women (82.6%), middle-aged (M=51.6), and children of the PwD (70.0%). The baseline protocol was completed by 52.9% of IC. Baseline participation rate was higher among spouses/children of PwD compared to other IC (46.5% vs. 27.5%; χ²(1, N=253)=4.194, p=.041). Compared to caregivers lost to follow-up, completers (65.3%) were significantly older (M 54.5 vs. M 46.7, t(174) =−4.220, p< 0.001), more often male (89.7% vs. 60.3%; χ²(1, N=175)=7.950, p=.005), children/spouses of PwD (69.9% vs. 34.8% others, χ²(1, N=176)=9.413, p=.002), cohabitating with PwD (82.4% vs. 60.0%, χ²(1, N=118)=6.197, p=.013), and had cared longer (Mdn 43 vs. Mdn 18; U=847, p=.008). The institutionalization rate was 7.8% at 9 months. iSupport-Portugal is a pioneering e-program exploited as an RMT that enabled cost-effective data collection from IC across national territories. High loss to follow-up calls for innovative retention strategies, especially for younger IC, which may intersect with platform user experience. Funding:FCT (2022.07587.PTDC)

